# 3D cellular visualization of intact mouse tooth using optical clearing without decalcification

**DOI:** 10.1038/s41368-019-0056-z

**Published:** 2019-08-27

**Authors:** Sujung Hong, Jingu Lee, Jin Man Kim, Sun-Young Kim, Hyung-Ryong Kim, Pilhan Kim

**Affiliations:** 1 Graduate School of Nanoscience and Technology, Korea Advanced Institute of Science and Technology (KAIST), 291 Daehak-ro, Yuseong-gu, Republic of Korea; 2 KI for Health Science and Technology (KIHST), Korea Advanced Institute of Science and Technology (KAIST), 291 Deahak-ro, Yuseong-gu, Republic of Korea; 3 Department of Dentistry, CHA Bundang Medical Center, CHA University, Seongnam, Republic of Korea; 4 Department of Conservative Dentistry and Dental Research Institute, Seoul National University School of Dentistry, 101 Daehak-ro, Jongno-gu, Republic of Korea; 5 College of Dentistry, Institute of Tissue Regeneration Engineering (ITREN), Dankook University, Cheonan, Republic of Korea; 6 Graduate School of Medical Science and Engineering, Korea Advanced Institute of Science and Technology (KAIST), 291 Daehak-ro, Yuseong-gu, Republic of Korea

**Keywords:** Dentistry, Dental pulp, Confocal microscopy, Fluorescence imaging, Pulpitis

## Abstract

Dental pulp is composed of nerves, blood vessels, and various types of cells and surrounded by a thick and hard enamel-dentin matrix. Due to its importance in the maintenance of tooth vitality, there have been intensive efforts to analyze the complex cellular-level organization of the dental pulp in teeth. Although conventional histologic analysis has provided microscopic images of the dental pulp, 3-dimensional (3D) cellular-level visualization of the whole dental pulp in an intact tooth has remained a technically challenging task. This is mainly due to the inevitable disruption and loss of microscopic structural features during the process of mechanical sectioning required for the preparation of the tooth sample for histological observation. To accomplish 3D microscopic observation of thick intact tissue, various optical clearing techniques have been developed mostly for soft tissue, and their application for hard tissues such as bone and teeth has only recently started to be investigated. In this work, we established a simple and rapid optical clearing technique for intact mouse teeth without the time-consuming process of decalcification. We achieved 3D cellular-level visualization of the microvasculature and various immune cell distributions in the whole dental pulp of mouse teeth under normal and pathologic conditions. This technique could be used to enable diverse research methods on tooth development and regeneration by providing 3D visualization of various pulpal cells in intact mouse teeth.

## Introduction

Teeth are highly structured tissues comprised of enamel, dentin, and pulp tissues. A mineralized hard tissue, the enamel-dentin matrix, surrounds the dental pulp, which is a soft connective tissue that includes blood vessels, nerves, and interstitial fluid with various pulpal cells. Pulpal cells include dental pulp stem cells (DPSCs), odontoblasts, fibroblasts, endothelial cells surrounded by pericytes forming vascular networks, and various immune cells including macrophages, granulocytes, mast cells, and plasma cells.^1–3^ In particular, odontoblasts, which are differentiated from DPSCs and located at the pulp-dentin interface, perform the specialized function of dentin maintenance by controlling the mineralization of reactive dentin and represent the first barrier against pathogens.^4,5^

Teeth are always in danger of decay by pathogens and metabolic products from various oral bacteria.^6^ When dental caries diffuse from the outer enamel to the pulp-dentin interface, resident odontoblasts activated by Toll-like receptors can recognize pathogens and secrete proinflammatory cytokines and chemokines. Then, innate immune responses to infection can be initiated with the accumulation of antigen-presenting dendritic cells, which are composed of resident macrophages and macrophages newly differentiated from monocytes; eventually, the carious sites can be repaired by removing the injurious agents.^7–9^ If uncontrolled inflammation continues and the infection deepens to the whole pulp and periapical region, a necrotic state causes permanent loss of function in the tooth.^10,11^ In addition, the infection of one tooth can be transferred to neighboring periodontal tissues due to the existence of microcirculation. This dental microcirculation is located through the lateral canals of the teeth and is related to the lymphatic and vascular systems; however, the presence of lymphatic vessels in mouse teeth is still a controversial issue.^12–15^

Because of the complexity of the dental pulp and its significance in tooth integrity, there have been extensive efforts to investigate various aspects of the dental pulp at the cellular and molecular levels. However, observation of the dental pulp at a microscopic scale has been a difficult task because the thick inorganic matrix surrounding the dental pulp blocks the penetration of the optical beam for imaging. For histological observation of a sectioned tooth, a long period of decalcification is an essential step before sectioning the sample.^16^ Decalcification to eliminate calcium ions is performed by using decalcifying agents that have the characteristics of acid-chelating agents such as formic acid-formalin and formalin-EDTA.^17,18^ This decalcification step inevitably hampers the evaluation of tooth mineralization in various situations, under both healthy and pathological conditions. Nevertheless, decalcified tooth becomes a soft gelatinous substance, and thin slices for microscopic observation can be obtained by mechanical sectioning. However, these conventional approaches based on thin sections of tissues have limitations in the analysis of the 3D cellular-level internal structure of the whole tissue due to the disruption of structural features, such as vacuolation and changes in the diameter of the dentinal tubules, during the process of decalcification and mechanical cutting of the tissue.^19^

To overcome this limitation, various tissue optical clearing techniques have been actively developed to analyze the complicated 3D cellular-level structure of thick tissue samples without the sectioning procedure.^20^ Tissue optical clearing techniques have been used for various soft tissues, such as the brain, colon, lymph node, and skin.^21–23^ As tissue optical clearing techniques have advanced, they have also been applied to hard tissues. In particular, using several different kinds of clearing methods,^24–27^ successful optical clearing and 3D imaging of mouse hard tissue, including the bone, mandible, and molar, have been reported. In most cases, however, a decalcification process is required to see the hard tissue, and as mentioned earlier, this approach is complex and time-consuming. In addition, it can disrupt tissue integrity and more importantly, hamper conventional methods to analyze tooth mineralization at the microscopic level. Interestingly, a modified Murray’s clear method using peroxide-free BABB (1:2 = benzyl alcohol:benzyl benzoate, RI = 1.559) was shown to have potential for the optical clearing of hard tissues such as bone and bone marrow without the process of decalcification.^28^ This method is a solvent-based clearing method that involves immersing a thick tissue sample in a specialized optical clearing agent (OCA) that has a high refractive index of >1.5 and good permeability into the thick tissue. This approach homogenizes the refractive indices of the cellular substances within the tissue and minimizes optical scattering, reducing light scattering, and enabling a ballistic light beam to deeply penetrate into the tissue.^29^ The modified Murray’s clear method using peroxide-free BABB has the advantages of being a simple procedure and having a short clearing time. However, its applicability to optical clearing for intact teeth has not yet been investigated. Therefore, we tried to use this rapid, simple, and inexpensive method for the optical clearing of intact teeth.

In this work, we established a modified Murray’s clear method optimized for the optical clearing of mouse teeth to achieve 3D cellular-level visualization. Using the established method, we successfully visualized the whole microvasculature in the dental pulp of an intact mouse tooth after systemic fluorescence labeling. In addition, the 3D distribution of immune cells expressing CD11c-YFP or CX3CR1-GFP in the dental pulp, the recruitment of granulocytes, and the vascular changes in a carious tooth were successfully visualized at the cellular level.

## Results

### 3D visualization of the dental pulp microvasculature in an optically cleared tooth

**Fig. 1:**
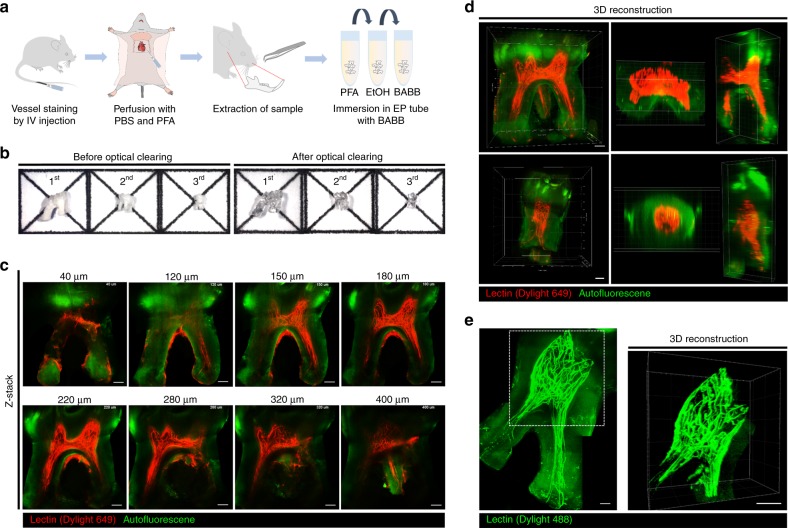
3D visualization of the dental pulp microvasculature in optically cleared teeth. **a** Schematic illustration of the optical clearing process for teeth for 3D visualization of the microvasculature: blood vessel staining, transcardial perfusion, tooth extraction, fixation with PFA, dehydration with EtOH, and immersion in the optical clearing agent BABB. **b** Photographs of teeth before and after optical clearing. **c** Z-confocal images of an upper second molar showing blood vessels (red) stained by Dylight 649-conjugated tomato lectin and autofluorescence (green) (Supplementary Video 1). **d** 3D reconstructed image of the microvasculature in an upper second molar and third molar rendered by using the Z-stack imaging data from **c** (Supplementary Video 2). **e** Maximal intensity Z-projection image of an upper third molar showing blood vessels (green) stained by Dylight 488-conjugated tomato lectin and autofluorescence (green), and 3D reconstruction image of the microvasculature in the dotted square (Supplementary Video 3). Scale bar, **c**–**e** 100 μm

We used the modified Murray’s clear method using peroxide-free BABB for the optical clearing of intact mouse teeth. First, molars extracted from wild-type C57BL/6N mice intravenously injected with far-red fluorescent Dylight 649-conjugated tomato lectin for fluorescence labeling of blood vessels were processed as illustrated in Fig. 1a. All three upper molars were successfully cleared to become optically transparent, as shown in Fig. 1b. Although there was some variation in the intensity of the green autofluorescence of the enamel and dentin matrix, we could robustly achieve successful optical clearing of the extracted tooth by following the procedure described in the “Methods” section. Interestingly, the enamel layer had much stronger autofluorescence than the dentin matrix. Because the strong autofluorescence of the enamel layer hampered the delivery of the excitation laser beam, side-view imaging of the dental pulp from the side of the optically cleared tooth bypassing the enamel layer was more appropriate than top-view imaging from the top of the tooth. The effective immersion period with peroxide-free BABB for the extracted mouse tooth was determined as 1 day by comparing Z-stack confocal imaging results of teeth immersed for 1–4 days. With Z-stack confocal imaging from the side of the optically cleared upper second molar, we successfully visualized the microvasculature in the dental pulp at a depth of >400 μm, as shown in Fig. 1c and Supplementary Video 1. In addition, the 3D rendered image reconstructed with the Z-stack imaging data showed a dense network of capillaries in the dental pulp, as shown in Fig. 1d and Supplementary Video 2. Although some of the vessels in one of the root canals were disconnected during the extraction process, we could visualize the whole microvasculature inside the dental pulp from the smaller third molar extracted from a wild-type mouse intravenously injected with green fluorescent Dylight 488-conjugated tomato lectin, as shown in Fig. 1e and Supplementary Video 3.

### 3D visualization of the immune cell distribution in an optically cleared tooth

To further extend the applicability of the established optical clearing methods, we investigated the efficacy of the procedure for teeth extracted from various transgenic mice expressing fluorescent proteins in specific types of immune cells. As a first step, we evaluated the degree of quenching of green fluorescent protein (GFP) with different concentrations of ethanol for dehydration, from 50 to 100%, followed by a 1-day immersion with BABB. The optimal concentration of ethanol was determined to be 80% by comparing the Z-stack imaging data, which showed the maximum fluorescence intensity of Dylight 649 at the same imaging depth with minimal quenching of the GFP fluorescence, as shown in Supplementary Fig. 1.

**Fig. 2:**
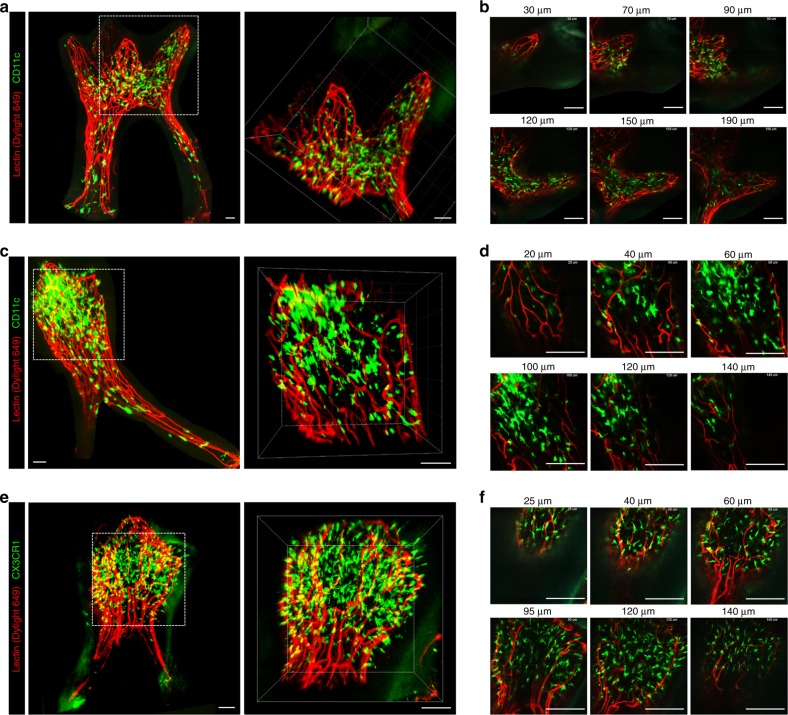
3D visualization of immune cell distributions in the dental pulp. **a** Maximal intensity Z-projection image of an upper second molar extracted from a CD11c-YFP transgenic mouse, with YFP-expressing CD11c + cells (green) and blood vessels (red). 3D reconstructed image in the dotted square in the dental crown (Supplementary Video 4). Scale bar, 50 μm. **b** Z-stack confocal images in the dotted square in **a**. Scale bar, 100 μm. **c** Maximal intensity Z-projection image of an upper third molar extracted from a CD11c-YFP transgenic mouse, with CD11c + cells (green) and blood vessels (red). 3D reconstructed image in the dotted square in the dental crown. Scale bar, 50 μm. **d** Z-stack confocal images in the dotted square in **c** (Supplementary Video 5). Scale bar, 100 μm. **e** Maximal intensity Z-projection image of an upper third molar extracted from a CX3CR1-GFP transgenic mouse, with GFP-expressing CX3CR1 + cells (green) and blood vessels (red). 3D reconstruction image in the dotted square (Supplementary Video 6). Scale bar, 50 μm. **f** Z-stack confocal images in the dotted square in **e**. Scale bar, 100 μm

The upper second and third molars extracted from CD11c-YFP transgenic mice expressing yellow fluorescent protein (YFP) in dendritic cells^30,31^ were optically cleared by following the previously described protocol. The 3D distribution of CD11c-expressing immune cells residing in the dental pulp of the intact molars was successfully visualized, as shown in Fig. 2a–d and Supplementary Videos 4 and 5. In addition, the upper third molar extracted from a CX3CR1-GFP transgenic mouse expressing GFP in monocytes and macrophages and in a subset of dendritic cells^32,33^ was optically cleared and imaged to visualize the 3D distribution in the dental pulp, as shown in Fig. 2e, f and Supplementary Videos 6 and 7. Both CD11c-YFP- and CX3CR1-GFP-expressing cells were mostly located in the pulp of the dental crown rather than in the root canal.

### 3D visualization of granulocyte recruitment and microvasculature changes in inflamed teeth

**Fig. 3:**
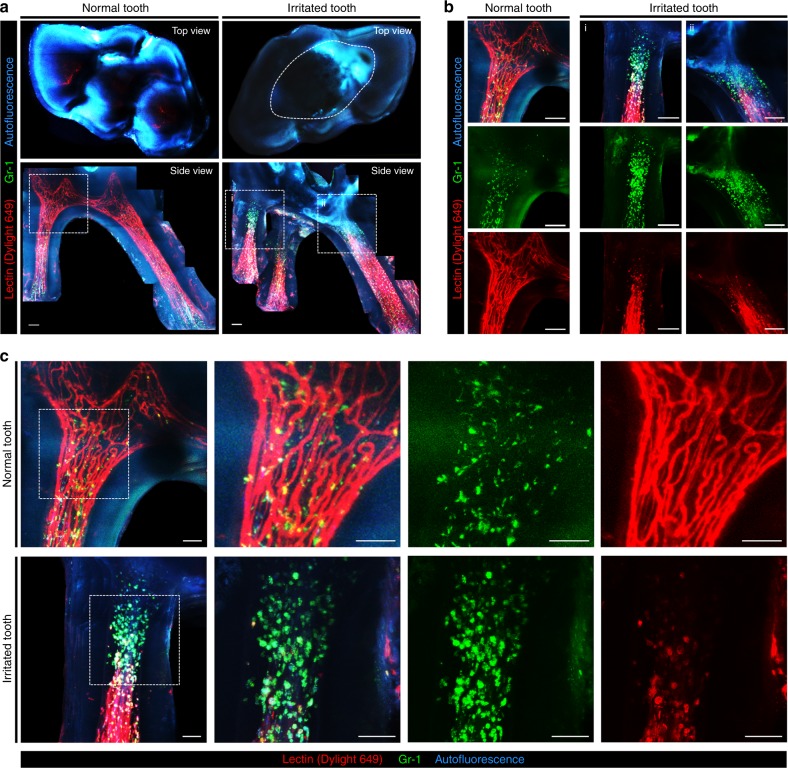
3D visualization of granulocyte recruitment and microvasculature changes in the dental pulp of an inflamed mouse tooth. **a** Top-view and side-view maximal intensity Z-projection image of an extracted normal tooth and a tooth irritated by mechanical burring; blood vessels (red), Gr-1 + cells (green), and autofluorescence (cyan). The burred area was delineated by a dashed line in the top-view image. Scale bar, 100 μm. **b** Magnified images in dotted squares in **a**. Scale bar, 100 μm. **c** Magnified images in dotted squares in **a** highlight changes in the granulocyte distribution and microvasculature in the irritated tooth. Scale bar, 50 μm

Dental pulpitis was induced by irritating the top of the molars with a drill bur. Two days after burring, the molars were extracted and optically cleared. Before extraction, blood vessels and recruited granulocytes in the dental pulp were fluorescently labeled by intravenous injection of tomato lectin and anti-Gr-1 antibody conjugated with a fluorophore, respectively. A normal tooth was collected as a control for comparison with the irritated tooth. Figure 3a shows the top-view and side-view maximal intensity projection image of the extracted normal tooth and irritated tooth. In the top view, the burred area is delineated by a dashed line. In the normal tooth, as previously shown in Figs. 1 and 2, a dense network of blood vessels was observed throughout the whole dental pulp from the crown to the root canal. In contrast, the blood vessels in the pulp of the irritated tooth were significantly regressed from the dental crown to the root. In the normal tooth, Gr-1-positive granulocytes were mostly located in the perivascular area and uniformly distributed throughout the dental pulp, as shown in Fig. 3a, b. In the inflamed tooth, an increased number of Gr-1-positive granulocytes was observed at the front end of the regressed blood vessels, as shown in Fig. 3b, suggesting the active recruitment of granulocytes through the blood vessels as an inflammatory response triggered by mechanical burring. In addition, a small portion of Gr-1-positive granulocytes observed in the dental pulp of the inflamed tooth were colocalized with lectin, as shown in Fig. 3c. This colocalization might be the result of phagocytic uptake of the lectin by granulocytes, which leaked from the blood vessel due to compromised integrity caused by the inflammatory response. In contrast, in the normal tooth shown in Fig. 3c, no granulocytes colocalized with lectin because the vessel integrity was maintained, and the intravenously injected lectin was confined inside the blood vessels.

## Discussion

In this work, we used a solvent-based optical clearing technique, modified Murray’s clear, for 3D cellular-level visualization of the dental pulp in intact mouse teeth. Using the modified Murray’s clear method with peroxide-free BABB solution, an intact tooth could be optically cleared by simple immersion for 1 day, as shown in Fig. 1a, b, without the time-consuming decalcification step, which has a processing time of 4–15 days. After optical clearing, the 3D microvasculature in the dental pulp was successfully visualized at the cellular level by acquiring Z-stack images with a custom-built laser-scanning confocal microscope shown in Fig. 1c–e. Blood flow in the pulpal tissues plays a critical role in maintaining tooth vitality by supplying essential biological factors. In pathological conditions, uncontrolled blood flow caused by the disruption of the vascular system increases the hydrostatic pressure in the dental pulp, resulting in tooth pain. Despite this importance, there has been a lack of useful methods for investigating the vascular system with integrated 3D structures. Therefore, comprehensive 3D visualization of the vasculature in an intact tooth, as demonstrated in this work, can be a useful method to analyze vascular changes in the dental pulp under physiologic and pathologic conditions. Furthermore, bypassing the decalcification step provides the potential utility of classical methods for evaluating the mineralization of the teeth after optical clearing. For example, microCT imaging to quantify mineralization could be performed on the same tooth sample in conjunction with cellular-level 3D visualization. This combinatorial strategy can enable accurate mapping of the spatial relationship between pathological changes in dentin structure and cellular status. In the same context, this approach can be applied to evaluate the biological response upon treatment with acidic restorative materials inducing decalcification or to provide additional information about the biomechanical properties of teeth correlated with cellular-level changes. Notably, avoiding decalcification is particularly effective for maintaining the integrity of peri- and intradentinal organic components such as collagen, growth factors, and peptides.^34^ Integrated collagen and mineralized dentin matrices are advantageous for preserving mechanical properties (e.g., microhardness and elastic modulus).^35^ These studies could be further explored in more detail by utilizing the developed methods of tooth optical clearing without decalcification.

To further optimize the clearing process to minimize the quenching of endogenous fluorescent proteins in the teeth optically cleared with BABB, we performed a quenching test with teeth extracted from a transgenic CX3CR-1-GFP mouse after systemic fluorescence labeling of vascular endothelial cells with the organic dye Dylight 649. We observed that the quenching of GFP in the dental pulp by the clearing process is more critically affected by the concentration of EtOH used for dehydration than the immersion time in BABB. To simultaneously assess the clearing efficacy and the quenching effect, confocal Z-stack imaging data were obtained from teeth treated with different concentrations of EtOH, 70%, 80%, and 100%, as shown in Supplementary Fig 1a. The average fluorescence intensity of GFP and Dylight 649 at different imaging depths was quantified, as shown in Supplementary Fig 1b. The fluorescence intensities of GFP in the dental pulp after optical clearing with 80% or 70% EtOH were similar at most of the imaging depths, but they were significantly higher than that in the tooth dehydrated with 100% EtOH. On the other hand, the fluorescence intensity of the organic dye Dylight 649 was highest in the tooth dehydrated with 80% EtOH at all of the imaging depths. Based on these observations, we determined that 80% was the optimal concentration of EtOH in the modified Murray’s clear method for tooth optical clearing bypassing the decalcification step and for subsequent 3D cellular-level imaging. Nevertheless, using 80% EtOH could be a beneficial and balanced choice because some water molecules are necessary to maintain emission from most fluorescent proteins, and complete dehydration has been regarded as one of the major limitations in conventional solvent-based clearing methods.^36^ In addition, it has been reported that GFP expression could be better sustained when the combination of aqueous solutions and solvents was used in comparison with the traditional BABB protocol using 100% EtOH, which is presumably due to the residual water in the samples.^36^ An alternative to using 80% EtOH is using tert-butanol for dehydration because it is less reactive than EtOH and more stable against oxidation.^37^ Indeed, there are several reports on using tert-butanol for improved preservation of fluorescent proteins in the process of optical clearing.^25,37–40^

Using the optimized protocol, we successfully visualized the 3D distribution of various immune cells expressing CD11c-YFP or CX3CR1-GFP in the dental pulp at the cellular level, as shown in Fig. 2. From the optically cleared upper molars extracted from the CX3CR1-GFP and CD11c-YFP mice, numerous immune cells with dendritic morphology were observed in the dental pulp. Using commercially available software, IMARIS, 3D surface rendering for the quantitative analysis of GFP-expressing cells and vasculature, such as automated cell counting or calculation of the vascular volume and density in the dental pulp, could be performed, as shown in Supplementary Video 7. Interestingly, the dendritic immune cells had a high level of morphological heterogeneity and were densely populated in the dental crown part of the pulp, as previously reported.^41,42^ In healthy human dental pulp, the expression of the chemokine receptor gene CX3CR1 has been reported to be extensively involved in cell-mediated immunity;^43^ however, its role in the immune response in caries lesions is mostly unknown. In addition, dendritic cells expressing a high level of CD11c have been identified in the dental pulp^31^ and confirmed to migrate to regional lymph nodes in response to exposure to cariogenic bacteria.^30^ Our optical clearing methods capable of comprehensive 3D visualization of dental pulp with fluorescent protein-expressing cells under the control of various promotors could be a useful tool to investigate the detailed roles of proteins of interest under both physiologic and pathologic conditions.

In addition, we visualized teeth with pulpitis by mechanical burring, as shown in Fig. 3a, b. In the inflamed pulp, an increased number of Gr1-positive immune cells were observed in the dental pulp near the exposed region, which presents a typical primary innate immune response triggered by exposure to external stimuli. In addition, no blood vessels were observed in the inflamed pulp of the crown part. In a carious tooth, because an inflammatory response is triggered by pathogen-associated molecular pattern molecules from cariogenic bacteria, expanded blood vessels with increased flow and permeability could increase the amount of interstitial fluids in the dental pulp. As a result, the increased interstitial pressure could induce vasoconstriction and eventually vascular necrosis in the pulp.^44–46^ Thus, regression of the blood vessel might be the result of necrosis of the blood vessels progressing from the damaged region.

To summarize, we optimized modified Murray’s clear, a solvent-based clearing technique, for simple and rapid optical clearing of intact teeth without decalcification. We successfully visualized the vasculature and immune cell distribution in the intact dental pulp from reporter mice with systemic fluorescence labeling and achieved 3D image reconstruction at the cellular level. The established technique could provide a comprehensive 3D cellular-level visualization of intact soft tissues surrounded by hard tissues where optical sources have difficulty in penetrating. Through further optimization of the presented optical clearing technique, it could become a versatile tool for the visualization of bone marrow systems in cancellous bone as well as dental pulp tissues in the teeth of various animal models. These advances could generate quantified spatiotemporal information for various pulpal cells (e.g., odontoblasts, DPSCs, neural cells, vascular cells, and fibroblasts) and bone marrow cells (e.g., hematopoietic stem cells, osteoblasts, and osteoclasts) in intact tissues with high resolution. Notably, DPSCs have been highlighted in several regenerative approaches for hard tissue reconstruction, such as dentinogenesis in the dentin-pulp complex^47^ and woven bone fabrication using hDPSCs,^48^ due to their potential in biomineralization and neovascularization. By enabling comprehensive 3D cellular-level visualization, the optical clearing method will be an efficient tool for monitoring the cell physiology of DPSCs during the regeneration process, especially during osteogenic or odontogenic differentiation. Therefore, our method could provide invaluable insights not only in dental science^49^ but also in other related regenerative research.^3,50,51^

## Materials and methods

### Animal model

Wild-type C57BL/6N mice were purchased from Orient Bio Inc. (Suwon, Korea). CX3CR1-GFP transgenic mice (Stock no. 005582) were purchased from Jackson Laboratory (Bar Harbor, USA). CD11c-YFP transgenic mice were generously provided by Dr. Choi at Hanyang University. All animal experiments were performed in accordance with the standard guidelines for the care and use of laboratory animals and were approved by the Institutional Animal Care and Use Committee (IACUC) of KAIST (protocol No. KA2017-36). All surgeries and procedures were performed under anesthesia, and all efforts were made to minimize animal suffering.

### Tooth sample

For 3D visualization of the microvasculature in dental pulp, teeth were dissected from a wild-type C57BL/6N mouse after systemic fluorescence labeling of endothelial cells in blood vessels via intravenous injection of Dylight 649-conjugated tomato lectin (DL-1178, Vector Laboratories) or Dylight 488-conjugated tomato lectin (DL-1174, Vector Laboratories). For 3D visualization of immune cells in the dental pulp, teeth were dissected from a CX3CR1-GFP mouse expressing GFP in mononuclear phagocytes^32^ and a CD11c-YFP mouse expressing GFP in dendritic immune cells and a subset of mononuclear phagocytes.^52^ Pulp inflammation was induced by making a cavity through the top of the molar using a drill bur, and the cavity was then immediately covered with capping material. At 2 days after burring, Dylight 649-conjugated tomato lectin and an anti-Gr-l (553122, BD biosciences) antibody conjugated with Alexa Fluor 555 (A20009, Invitrogen) were intravenously injected to fluorescently label blood vessels and granulocytes, respectively.

### Optical clearing procedure

Mice were anaesthetized with a mixture of Zoletil (30 mg/kg) and xylazine (10 mg/kg), and transcardial perfusion was performed with phosphate-buffered saline (PBS; LB004-02, Welgene) and 4% wt/vol paraformaldehyde (PFA; BPP-9016, T&I, and diluted in PBS). The teeth were extracted and washed with PBS for 1 min and then further immersed in 4% PFA at 4 °C for 1 day, followed by 80% wt/vol ethanol (EtOH CAS 64-17-5; 4022–4100, Daejung,and diluted in distilled water; DW) for 1 day at room temperature. As OCA, BABB solution was made by mixing a 1:2 vol:vol ratio of benzyl alcohol (402834, Sigma) and benzyl benzoate (B6630-1L, Sigma). Teeth dehydrated by ethanol were immersed in an EP tube with peroxide-free BABB, which was made by mixing 40 ml of BABB solution with 10 g of aluminium oxide (Al_2_O_3_; 199443, Sigma) and removing the supernatant after centrifuging the mixed solvents at 2 000 × *g* for 10 min.^28^ Then, the tube containing the teeth was fixed on a rotator and kept at room temperature for 1 day.

### Imaging system

To visualize the optically cleared tooth in 3D at the cellular level, a previously described custom-built laser-scanning confocal microscope^53–55^ was used. Three laser modules with wavelengths at 488 nm (MLD488, Cobolt), 561 nm (Jive, Cobolt), and 640 nm (MLD640, Cobolt) were utilized as excitation light sources. For laser scanning, a fast-rotating polygonal mirror with 36 facets (MC-5, aluminium coated, Lincoln Laser) and a galvanometer mirror scanner (60 H, Cambridge Technology) were used. To illuminate the optically cleared tooth with the two-dimensional raster scanning laser beam and collect fluorescence signals in an epi-detection manner, commercial objective lenses (CFI Plan Apo lambda, 10X, NA 0.45, Nikon; CFI Plan Apo lambda, 20X, NA 0.75, Nikon; and LUCPLFLN, 40X, NA 0.6, Olympus) were used. Three highly sensitive photomultiplier tubes (PMT; R9110, Hamamatsu) with bandpass filters (FF02-525/50, FF01-600/37, FF01-685/40, Semrock) were employed for detecting multicolor fluorescence signals. A three-channel frame grabber (Solios, Matrox) was used to acquire the voltage output of the photomultiplier. A custom-written software program based on the Matrox Imaging Library (MIL9, Matrox) was used for image acquisition.

### Image processing

ImageJ (NIH) was used to generate Z-projection images with brightness and contrast adjustment. The brightness/contrast tool of ImageJ (NIH) was used to reduce background noise in the acquired Z-stack imaging data to the minimal level of <4% of the maximal signal. No additional image filter was used to improve contrast. Z-projection images were generated from the adjusted Z-stack imaging data, from the Z-position at which the vessels or cells began to appear to the Z-position at which they became invisible. 3D reconstruction was conducted with IMARIS (Bitplane). The maximum image projection mode was selected for 3D volume rendering in surpass view, and the low signal value was adjusted with the display adjustment function. Categorization of cells and the vascular structure was visualized by using the surface detection tool. Individual cell distribution was analyzed by using the spot detection tool. In Supplementary Video 7, detailed parameters were sequentially adjusted for surface detection: surface area detail level = 1 μm (for cells and blood vessels; green and red channels), diameter of the largest sphere that fits into the object = 3.66 μm (for both channels), and manual threshold value = 84.397 6 (for cells; green channel) and 50.403 1 (for blood vessels; red channel). The spot detail parameter of estimated diameter was set as 7 μm (for cells; green channel).

## Supplementary information

Supplementary Material

Supplementary Figure 1

Supplementary Video 1

Supplementary Video 2

Supplementary Video 3

Supplementary Video 4

Supplementary Video 5

Supplementary Video 6

Supplementary Video 7
