# Supplementary material for: 3D cellular visualization of intact mouse tooth using optical clearing without decalcification
Source: Int J Oral Sci. 2019 Aug 27;11(3):25. doi: 10.1038/s41368-019-0056-z (PMC6802633; doi:10.1038/s41368-019-0056-z)

**Supplemental Materials for**

**3D cellular visualization of intact mouse tooth using optical clearing without decalcification**

**Sujung Hong^1,2^, Jingu Lee^1,2^, Sun Young Kim^4^, Hyung-Ryong Kim^5,*^, and Pilhan Kim^1,2,3*^**

^1^Graduate School of Nanoscience and Technology, Korea Advanced Institute of Science and Technology (KAIST), 291 Daehak-ro, Yuseong-gu, Daejeon, 34141, Republic of Korea

^2^KI for Health Science and Technology (KIHST), Korea Advanced Institute of Science and Technology (KAIST), 291 Deahak-ro, Yuseong-gu, Daejeon, 34141, Republic of Korea

^3^Department of Dentistry, CHA Bundang Medical Center, CHA University, Seongnam, 13496, Republic of Korea

^4^Department of Conservative Dentistry, Seoul National University School of Dentistry, 101 Daehak-ro, Jongno-gu, Seoul, 03080, Republic of Korea

^5^Institute of Tissue Regeneration Engineering (ITREN), Dankook University, Cheonan 330-714, Republic of Korea

^6^Graduate School of Medical Science and Engineering, Korea Advanced Institute of Science and Technology (KAIST), 291 Daehak-ro, Yuseong-gu, Daejeon, 34141, Republic of Korea

* [pilhan.kim@kaist.ac.kr](mailto:pilhan.kim@kaist.ac.kr), hrkimdp@gmail.com

**Supplemental Materials Contents**

Supplemental Figure 1
Supplemental Video 1-7

**
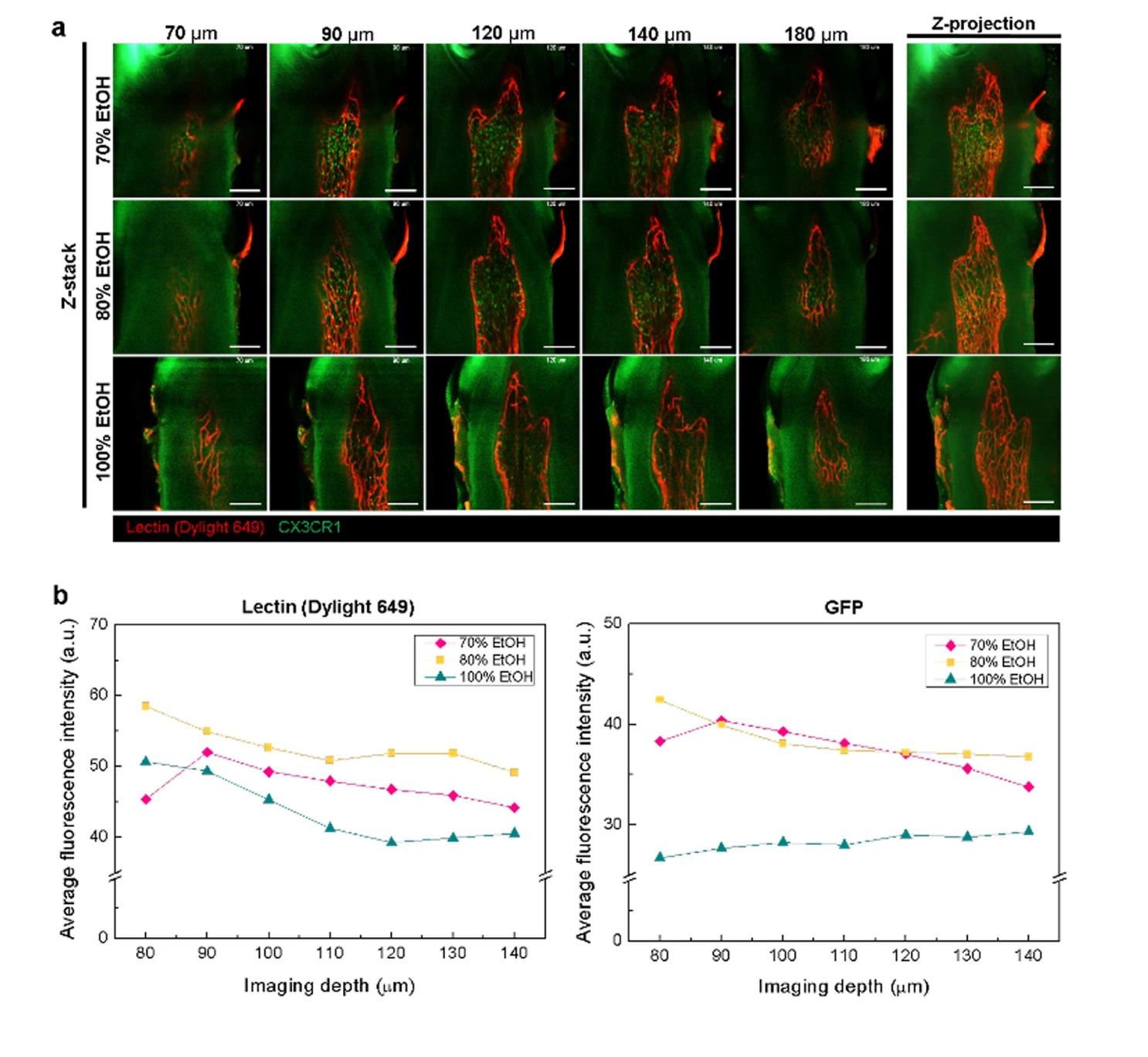
**

**Supplemental Figure 1. Comparison of the quenching of GFP and Dylight 649 with different concentration of EtOH**

(**a**) Z-stack confocal images of lower 3^rd^ molars extracted from CX3CR1-GFP transgenic mouse treated with different concentrations of EtOH, 70%, 80% and 100%, showing GFP expressing CX3CR1+ cells (green) and blood vessels (red) stained by Dylight 649 conjugated tomato lectin. Scare bar, 100 μm. (**b**) The average fluorescence intensity of GFP and Dylight 649 at different imaging depth in Z-stack confocal images.

**
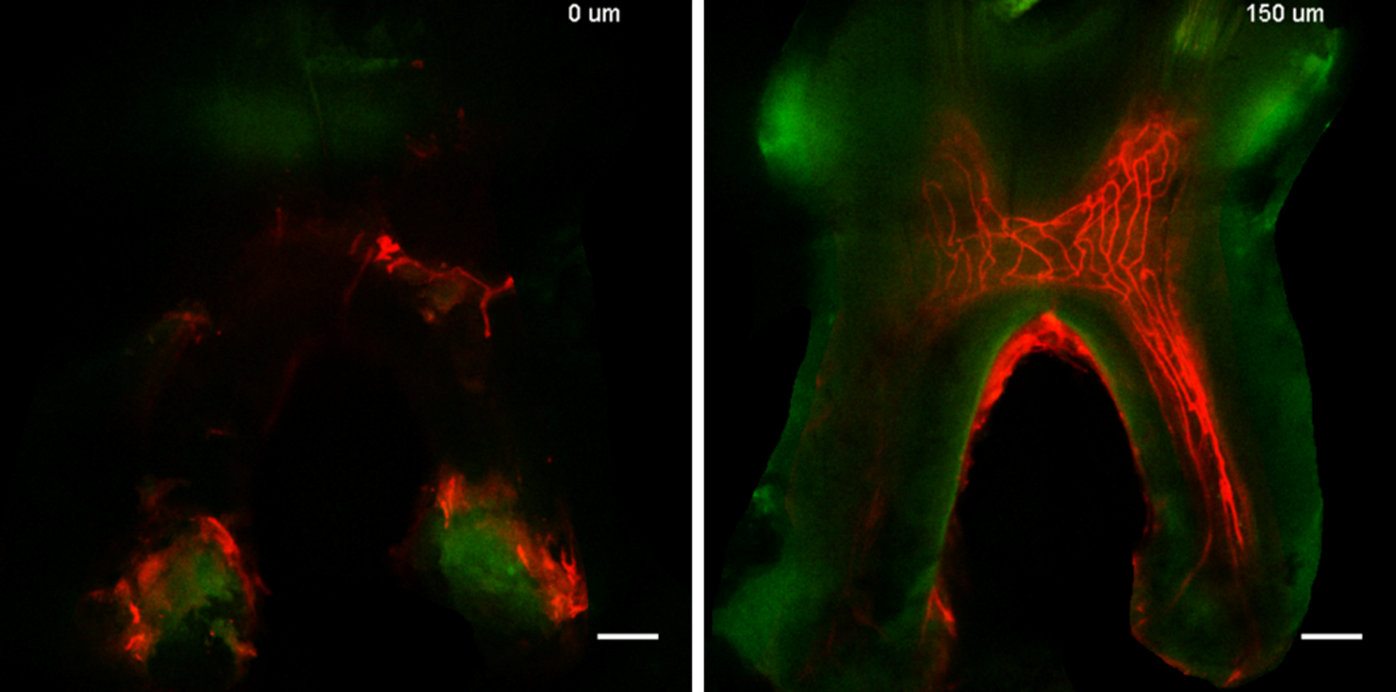
**

**Supplemental Video 1.** Z-stack imaging of optically cleared 2^nd^ molar of wildtype C57BL/6N mouse. Scale bar, 100 μm.

**
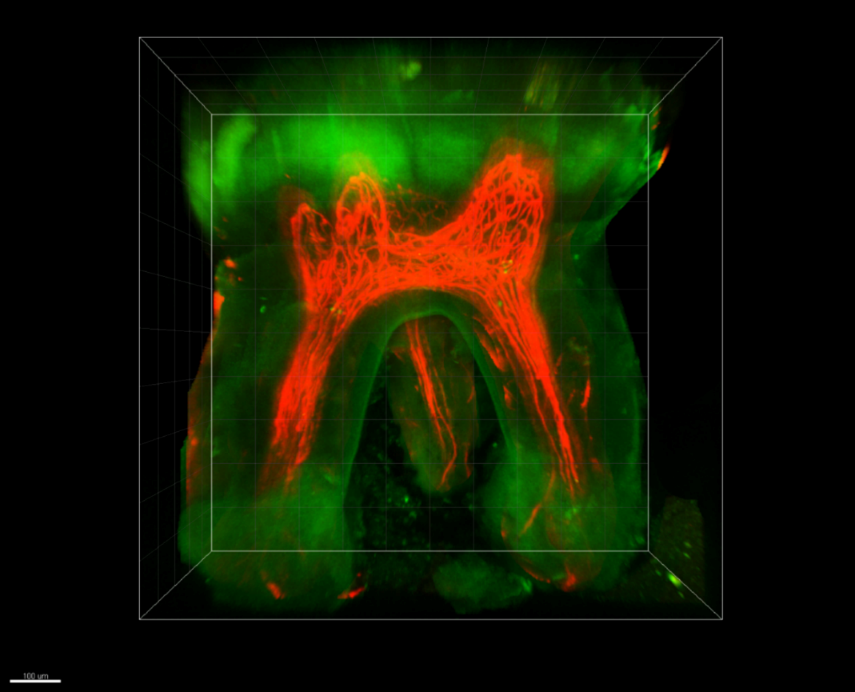
**

**Supplemental Video 2.** 3D reconstruction of optically cleared 2^nd^ molar of wildtype C57BL/6N mouse. Scale bar, 100 μm.

**
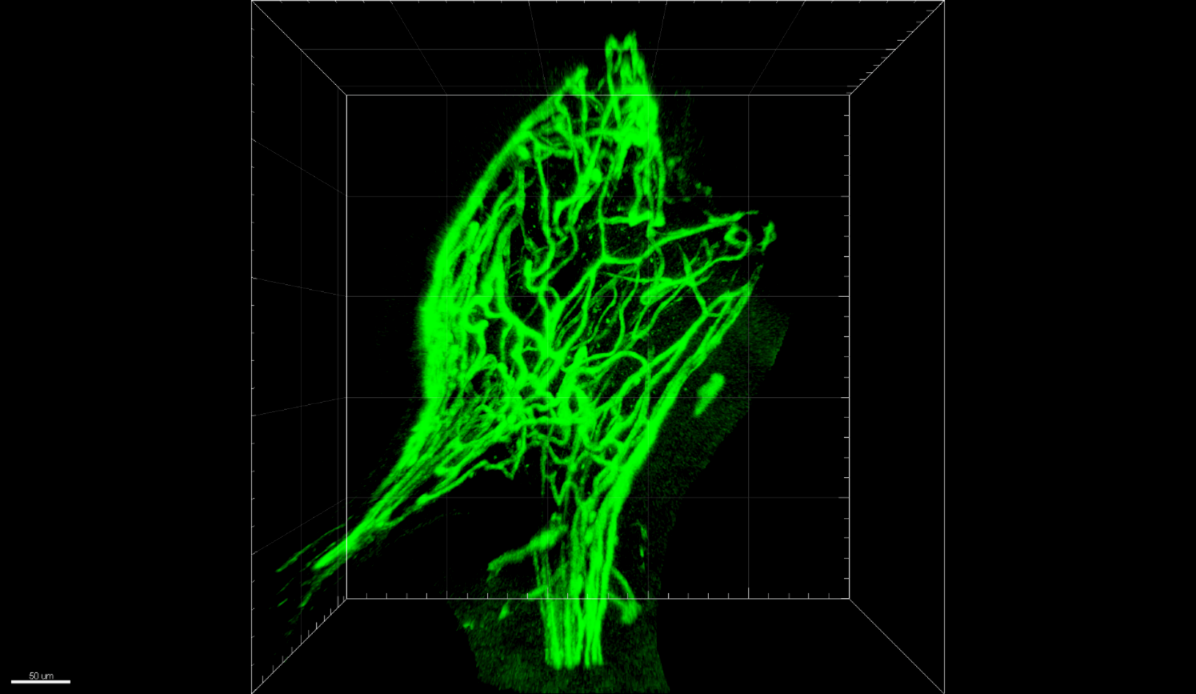
**

**Supplemental Video 3.** 3D reconstruction of optically cleared 3^rd^ molar of wildtype C57BL/6N mouse. Scale bar, 50 μm.

**
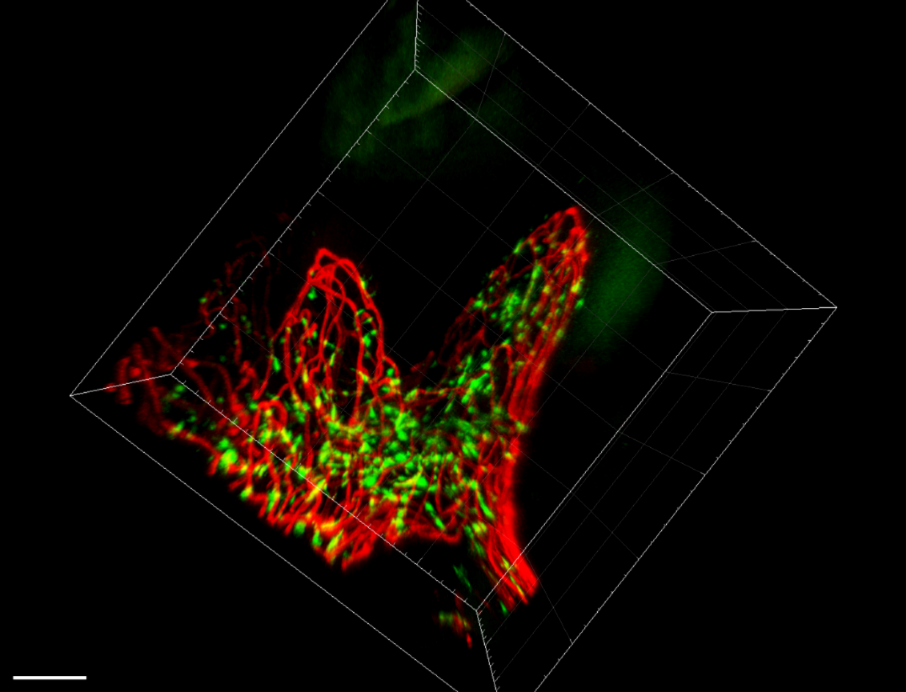
**

**Supplemental Video 4**. 3D reconstruction of optically cleared 2^nd^ molar of CD11c-YFP mouse. Scale bar, 50 μm.

**
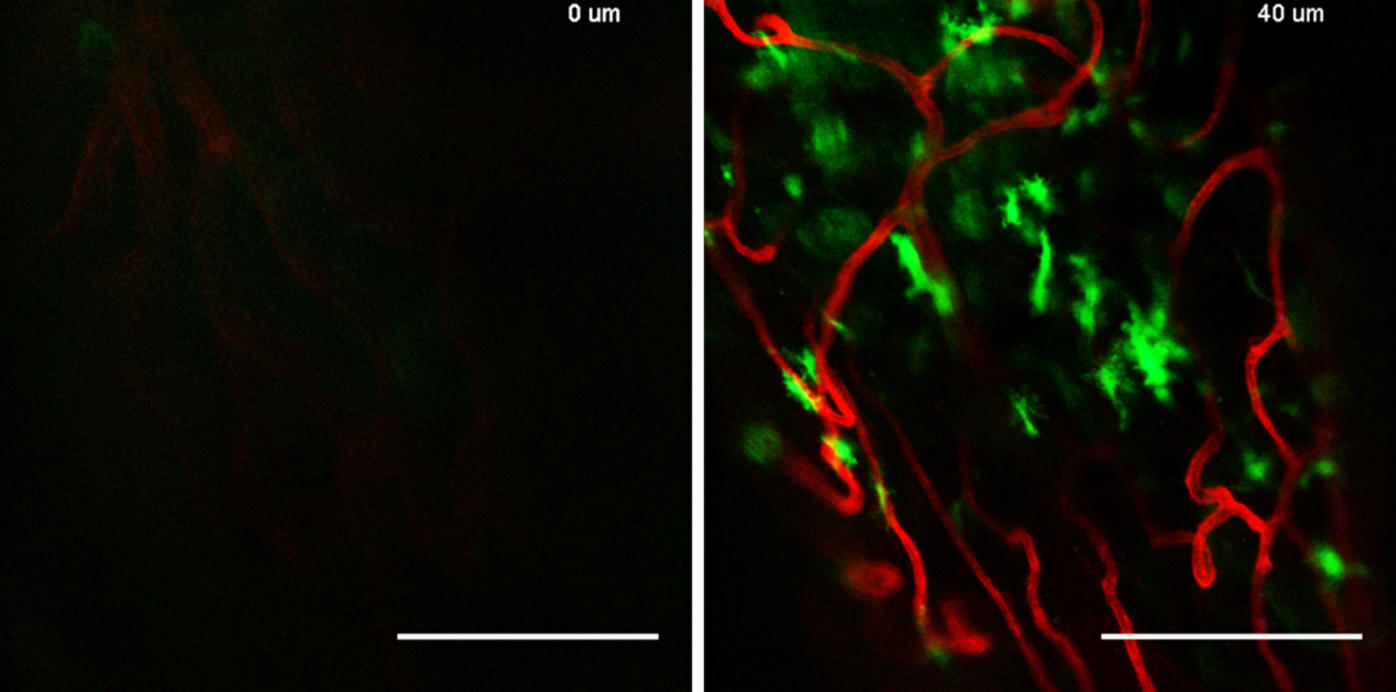
**

**Supplemental Video 5.** Magnified Z-stack imaging of optically cleared 3^rd^ molar of CD11c-YFP mouse. Scale bar, 100 μm.

**
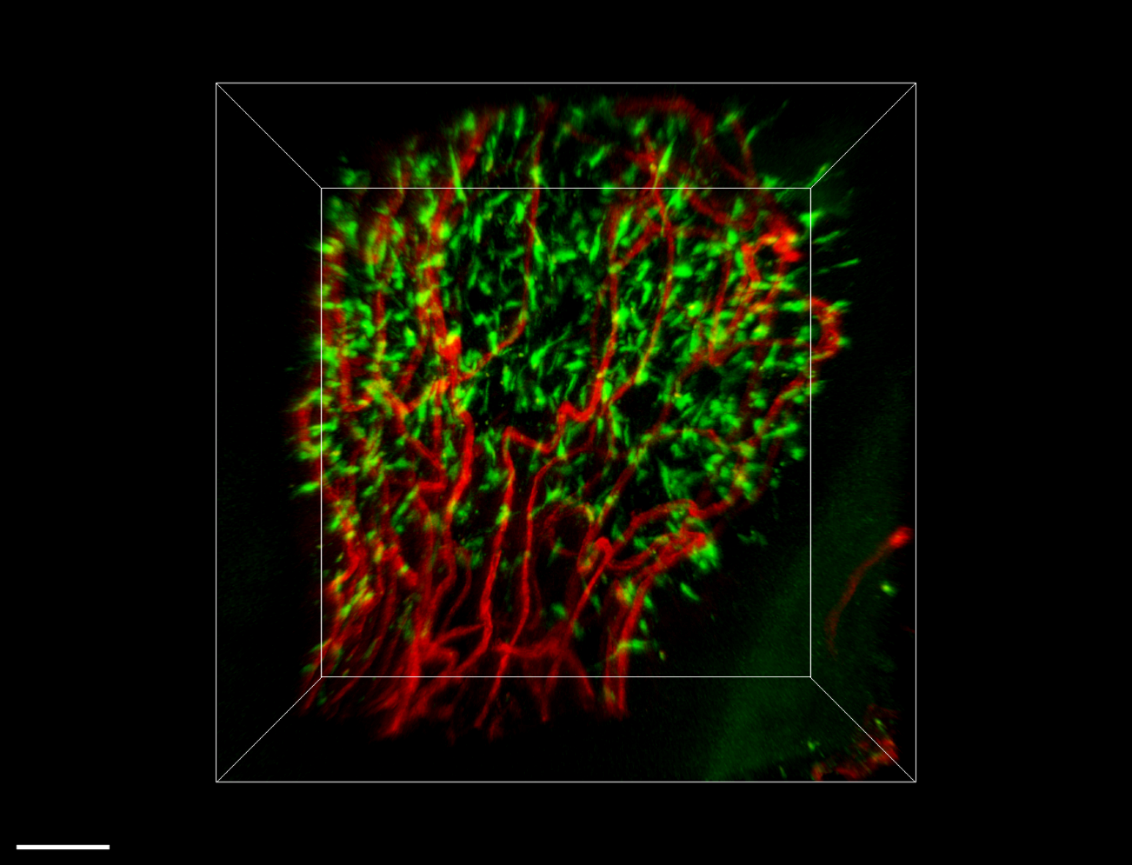
**

**Supplemental Video 6.** Magnified 3D reconstruction of optically cleared 3^rd^ molar of CX3CR1-GFP mouse. Scale bar, 50 μm.

**Supplemental Video 7.** 3D rendering and analysis of the distribution of CX3CR1-GFP cells and vasculature in dental pulp. Scale bar, 50 μm.
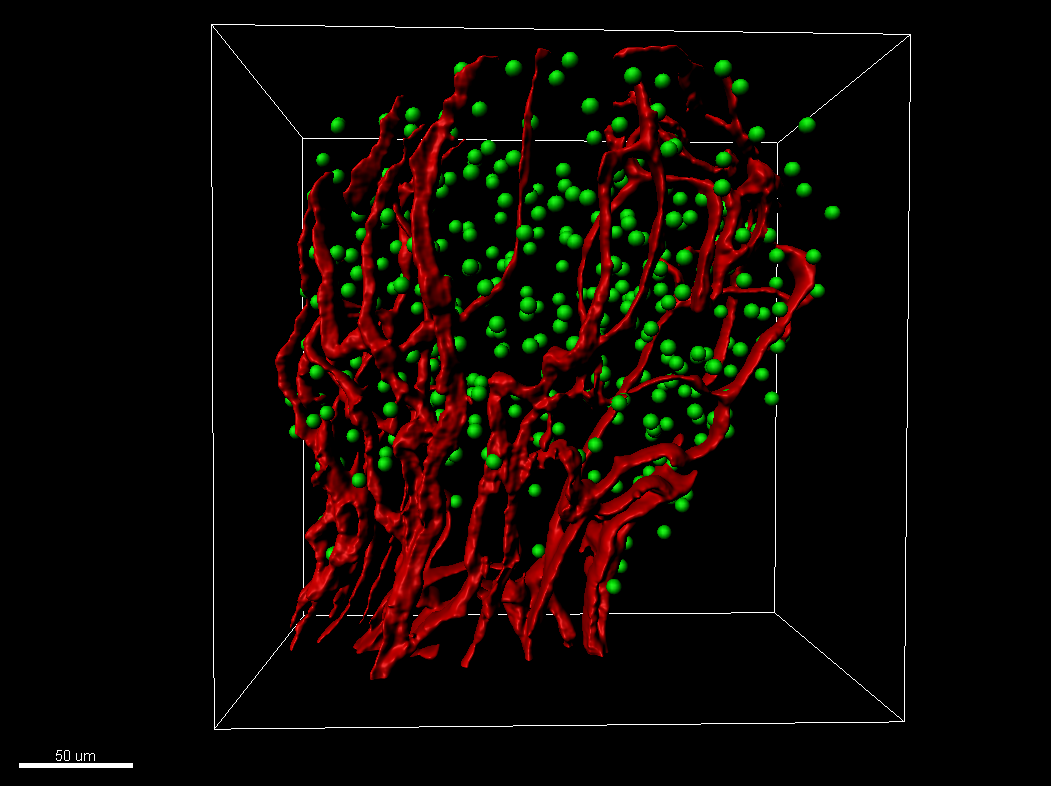

Supplement: Supplementary file 1 — Supplementary Material [file 41368_2019_56_MOESM1_ESM.docx]
