# Supplementary figures and images for: 3D cellular visualization of intact mouse tooth using optical clearing without decalcification
Source: Int J Oral Sci. 2019 Aug 27;11(3):25. doi: 10.1038/s41368-019-0056-z (PMC6802633; doi:10.1038/s41368-019-0056-z)

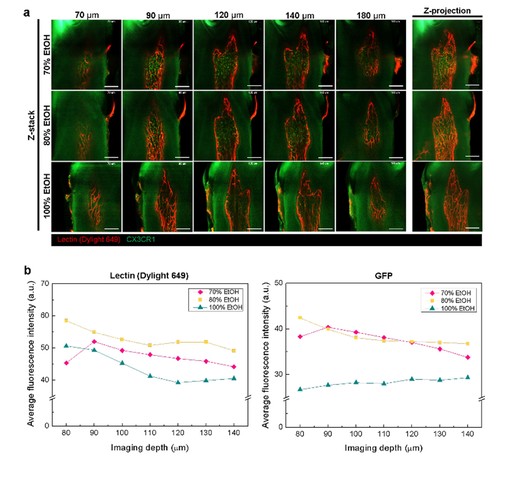

Supplement: Supplementary file 2 — Supplementary Figure 1 [file 41368_2019_56_MOESM2_ESM.jpg]
